# Investigating the diversity of bioluminescent marine worm *Polycirrus* (Annelida), with description of three new species from the Western Pacific

**DOI:** 10.1098/rsos.230039

**Published:** 2023-03-29

**Authors:** Naoto Jimi, Manabu Bessho-Uehara, Koji Nakamura, Masahiko Sakata, Taro Hayashi, Shusei Kanie, Yasuo Mitani, Yoshihiro Ohmiya, Aoi Tsuyuki, Yuzo Ota, Sau Pinn Woo, Katsunori Ogoh

**Affiliations:** ^1^ Sugashima Marine Biological Laboratory, Graduate School of Science, Nagoya University, 429-63 Sugashima, Toba, Mie 517-0004, Japan; ^2^ Centre for Marine & Coastal Studies, Universiti Sains Malaysia, 11800 USM Penang, Malaysia; ^3^ Institute for Advanced Research, Nagoya University, 464-8601 Nagoya, Japan; ^4^ Graduate School of Science, Nagoya University, 464-8601 Nagoya, Japan; ^5^ Japan Underwater Films Co., Ltd., 2-11-15, Nakaochiai, Shinjyuku, Tokyo 161-0032, Japan; ^6^ EVIDENT CORPORATION, Shinjuku Monolith, 3-1 Nishi-Shinjuku 2-chome, Shinjuku-ku, Tokyo, Japan; ^7^ Bioproduction Research Institute, National Institute of Advanced Industrial Science and Technology (AIST), Sapporo 062-8517, Japan; ^8^ Biomedical Research Institute, AIST, Ikeda 563-8577, Japan; ^9^ Graduate School of Science, Hokkaido University, Sapporo 060-0810, Japan; ^10^ San'in Kaigan Geopark Museum of the Earth and Sea, 1794-4, Makidani, Iwami-town, Tottori 681-0001, Japan; ^11^ HATENOURUMA, Hachioji, Tokyo 192-0023, Japan

**Keywords:** bioluminescence, Polychaeta, *Polycirrus aoandon* sp. nov., *Polycirrus ikeguchii* sp. nov., *Polycirrus onibi* sp. nov., terebelliformia

## Abstract

Bioluminescence, a phenomenon observed widely in organisms ranging from bacteria to metazoans, has a significant impact on the behaviour and ecology of organisms. Among bioluminescent organisms, *Polycirrus*, which has unique emission wavelengths, has received attention, and advanced studies such as RNA-Seq have been conducted, but they are limited to a few cases. In addition, accurate species identification is difficult due to lack of taxonomic organization. In this study, we conducted comprehensive taxonomic survey of Japanese *Polycirrus* based on multiple specimens from different locations and described as three new species: *Polycirrus onibi* sp. nov., *P*. *ikeguchii* sp. nov. and *P*. *aoandon* sp. nov. The three species can be distinguished from the known species based on the following characters: (i) arrangement of mid-ventral groove, (ii) arrangement of notochaetigerous segments, (iii) type of neurochaetae uncini, and (iv) arrangement of nephridial papillae. By linking the bioluminescence phenomenon with taxonomic knowledge, we established a foundation for future bioluminescent research development. We also provide a brief phylogenetic tree based on cytochrome c oxidase subunit I (COI) sequences to discuss the evolution of bioluminescence and the direction of future research.

## 1. Introduction

Bioluminescence is a well-documented phenomenon that occurs in many different organisms, including bacteria and fish, and has evolved independently in various lineages [1,2]. Bioluminescence has been reported in 14 annelid families, of which one is sipunculids [3], five are clitellates [4], and the rest are polychaetes [5–7]. Despite its occurrence, there is still much that is unknown about bioluminescence in polychaetes, including how it evolved and what it means ecologically. More research is needed to better understand this phenomenon, and to do this, we need to accumulate more examples of bioluminescent polychaetes [3,5,7–12].

One genus of polychaetes that has been the subject of recent taxonomic research is *Polycirrus* Grube, 1850 [13] [14–21]. Unlike other genera of Terebelliformia, the members of *Polycirrus* do not make solid tubes, and they are found in shallow to deep waters, inhabiting environments such as sandy to muddy sediments, maerl or seagrass beds, the interior of sponges and the cracks of rocky or coral substrates [18,21–23]. The classification of this genus and its ‘family’ has been the subject of much debate based on morphological and molecular data [21,24–26]. Since this study does not focus on higher-level classification, we will treat *Polycirrus* as a genus of Terebelliformia without mentioning its family level. As Lavesque [21] has noted, future studies will probably shed more light on the taxonomy of this group. Genera similar to *Polycirrus* are known to include *Amaeana* Hartman, 1959 [27], *Biremis* Polloni, Rowe and Teal, 1973 [28], *Enoplobranchus* Verrill, 1879 [29], *Hauchiella* Levinsen, 1893 [30] and *Lysilla* Malmgren, 1866 [31] and can be differentiated based on the presence or absence of notochaetae and neurochaetae, and the form of the neurochaetae [22]. This paper adheres to the diagnosis of the genus *Polycirrus* according to Glasby & Hutchings [32]. *Polycirrus* currently consists of 77 described species from all over the world [18,21,32]. In Japan, two species have been described from this genus: *P*. *nervosus* Marenzeller, 1884 [33] from the coast of Enoshima and *P*. *medius* Hessle, 1917 [34] from the subtidal area of the Sagami Sea [33,34]. It is also known that members of this genus have the potential for bioluminescence, but only four species have been documented as bioluminescent to date: *P*. *aurantiacus* Grube, 1860 [35] (Croatia), *P*. *perplexus* Moore, 1923 [36] (USA), *Polycirrus* sp. (Hawaii), and *Polycirrus* sp. (Japan) [37–40]. Huber *et al*. [39] reported for *Polycirrus perplexus* a peak emission of 445 nm, noting it as an exceptionally short wavelength for a coastal organism. In line with this, Kanie *et al*. [40] have recently reported that one unnamed Japanese species of *Polycirrus* emitted light at a wavelength of 444 nm. Further research into the unique luminescence of this genus could provide valuable insights into the mechanisms and evolution of luminescent phenomena.

In this study, we describe three bioluminescent species of the genus *Polycirrus* that were obtained from various parts of Japan. After conducting a thorough examination of the specimens based on morphological and genetic information, we concluded that they did not match to any of the known species in the genus, including the two species previously described from Japan. We therefore describe them as three new species: *Polycirrus aoandon* sp. nov., *Polycirrus ikeguchii* sp. nov. and *Polycirrus onibi* sp. nov. We also provide the DNA barcode of the cytochrome c oxidase subunit I (COI) region for these species and present a concise molecular phylogenetic relationship tree of the three new species. Additionally, we have recorded videos of the luminescence activities exhibited by these species of *Polycirrus*.

## 2. Material and methods

**Figure 1.**
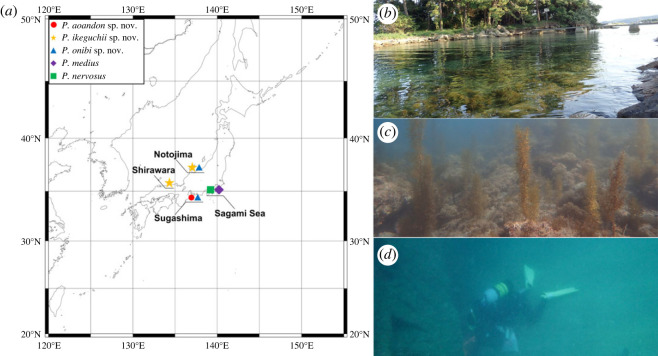
Sampling sites of *Polycirrus* spp. in this study. (*a*) Sampling locations and type localities of *P*. *medius* and *P*. *nervosus*; (*b*) Notojima, Ishikawa; (*c*) Sugashima, Mie; (*d*) off Shirawara Coast, Tottori.

*Polycirrus* worms were collected from various locations in Japan (figure 1) and examined for bioluminescence both in the field and in the laboratory [40]. The photographs and videos were taken with a digital camera (*α*7S, Sony, Tokyo, Japan) with a macro lens SEL50M28 (Sony) for laboratory observation, and with SEL24F18Z lens (Sony) with an underwater camera housing (Nauticam NA A7, Nauticam, Hong Kong, China) for *in situ* observation. The bioluminescence was stimulated by poking with tweezers in the laboratory or by air bubbling from SCUBA gear in the field. The sampling was conducted with the permission of the local government (permission no.: i-1010). The specimens were preserved in 70% ethanol and subsequently examined under stereomicroscopes (Nikon SMZ1500 and Nikon Ni-U). The specimens were then deposited in the National Museum of Nature and Science in Tsukuba (NSMT). In this study, we followed the morphological terminology of Glasby & Hutchings [32] and Lavesque *et al*. [21].

To obtain sequences in the barcoding region of the mitochondrial cytochrome c oxidase subunit I (COI) gene, DNA extraction, sequencing, alignment and removal of ambiguous positions were carried out using the methods described in Jimi *et al*. [41]. DNA was extracted from the tentacles of the holotype, and the newly obtained sequences were deposited in GenBank (Accession nos. OQ067377–OQ067380 (https://www.ncbi.nlm.nih.gov/genbank)). In addition to these sequences, additional sequences for other *Polycirrus* species were obtained from GenBank. A total of 23 sequences (for 21 species) were used in the phylogenetic analysis by MEGAX based on 657 bp of COI using maximum-likelihood method with a GTR + G evolutionary model [42]. The LSID for this publication is: urn:lsid:zoobank.org:pub:2C00A2F7-7CA1-490C-A2CA-20CB85AA80B0.

## 3. Results

### 3.1. Bioluminescent behaviour

**Figure 2.**
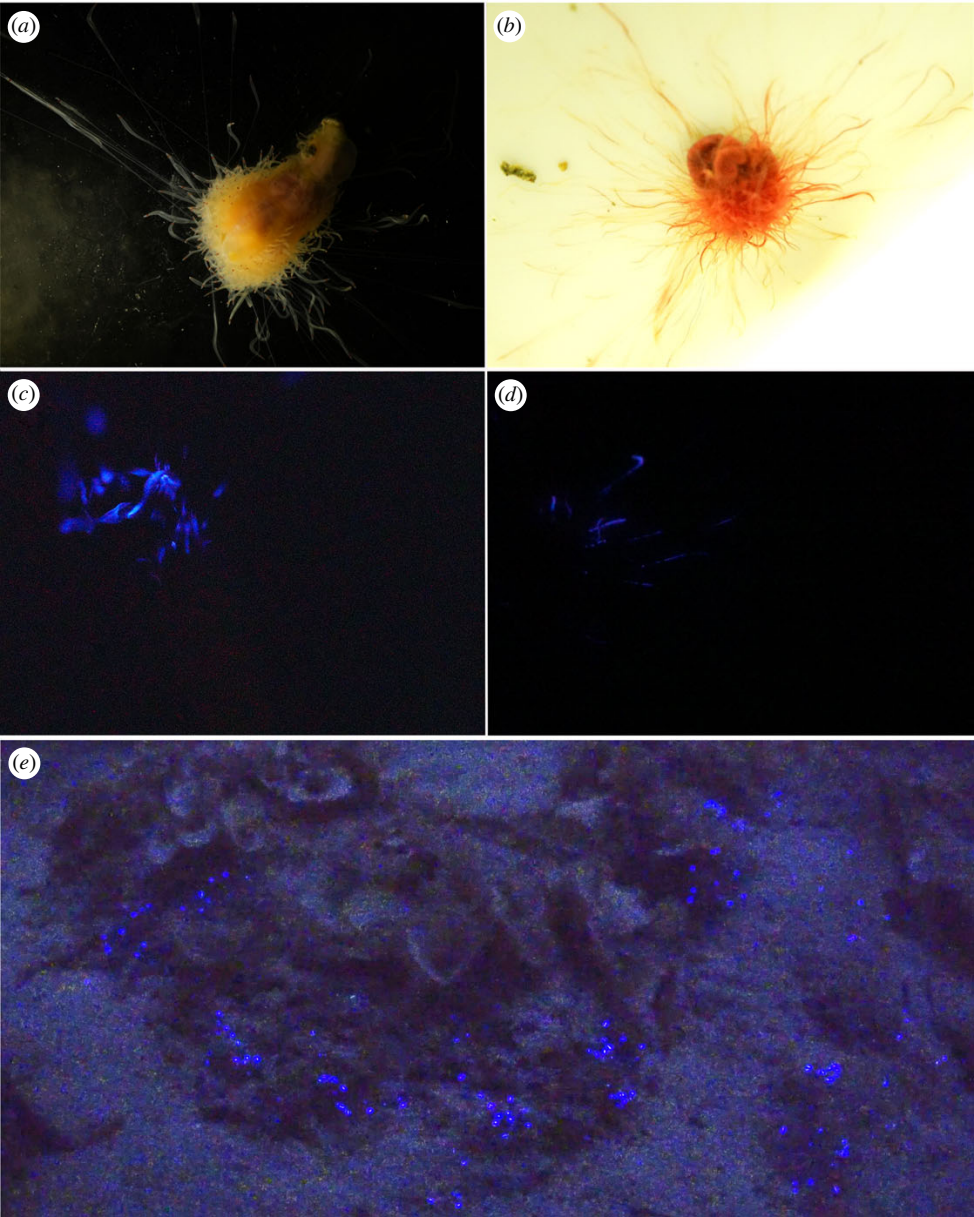
Bioluminescence of *Polycirrus* spp. (*a*,*c*) *Polycirrus onibi* sp. nov.; (*b*,*d*) *Polycirrus aoandon* sp. nov.; (*e*) *in situ* observation of *Polycirrus* spp. (mixture of *P*. *ikeguchii* sp. nov. and *P*. *onibi* sp. nov., most of them are *P*. *ikeguchii* sp. nov.) at Notojima.

At Sugashima Marine Biological Laboratory, we conducted an observation of the luminescent activity displayed by *Polycirrus onibi* sp. nov. and *P*. *aoandon* sp. nov. (figure 2*a–d*). Upon mechanical stimulation using tweezers, the tentacles of both species emitted an intense blue–purple luminescence. The stimulated tentacles flash (duration about 0.1 s) for a short period of time (0.3–1.1 s). The luminescent flashes were not found to trigger the neighbouring tentacles or to synchronize in an individual. Since we have only compared the luminescence of one individual from each species, we cannot draw definitive conclusions regarding the differences in the luminescence patterns between the two species. Electronic supplementary material, videos S1 and S2 provide further documentation of the luminescence of both species, and electronic supplementary material, video S3 has been provided for *in situ* observation of bioluminescence by SCUBA diving at Notojima (as depicted in figure 2*e*). The luminescence of *Polycirrus* in the colony was absent without bubbling stimulation, and it was evoked by bubbling air. The light intensity of the bioluminescence began to decrease after a continuous stimulation period of approximately 30 s. The capability of light emission was restored after a few minutes. Presumably, all the individuals in the colony are *P*. *ikeguchii*, and they emit approximately 0.1 s flash which repeats for 0.3–1.1 s. The bioluminescence of *P*. *ikeguchii* sp. nov. was also documented by Kanie *et al*. [40].

## 4. Systematics

### 4.1. Taxonomic account

Genus *Polycirrus* Grube, 1850 [13]

[New Japanese name: Hikari-Fusa-gokai-zoku]

*Type-species*. *Polycirrus medusa* Grube, 1850 [13].

*Polycirrus onibi* Jimi, sp. nov.

[Japanese name: Onibi-fusa-gokai]

(figures 2–4)

**Figure 3.**
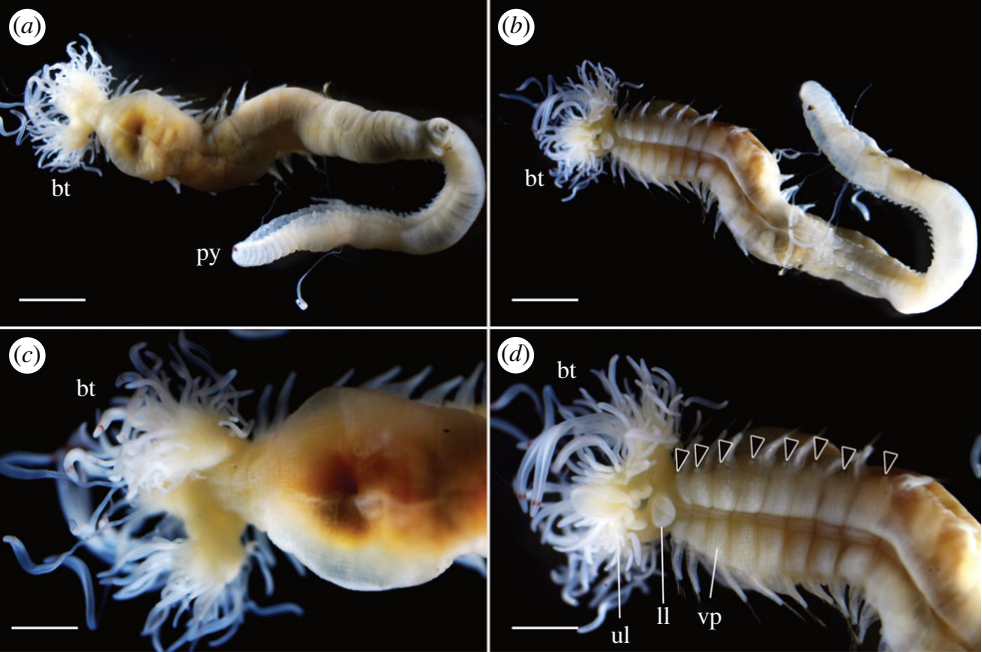
*Polycirrus onibi* sp. nov., holotype (NSMT-Pol H-913), fixed specimen. (*a*) Whole body, dorsal view; (*b*) whole body, ventral view; (*c*) anterior end, dorsal view; (*d*) anterior end, ventral view. Scale bars: (*a*,*b*) 2 mm; (*c*,*d*) 1 mm. bt, buccal tentacles; ll, lower lip; py, pygidium; ul, upper lip; vp, ventral pad. Arrow heads indicate nephridial papillae.

**Figure 4.**
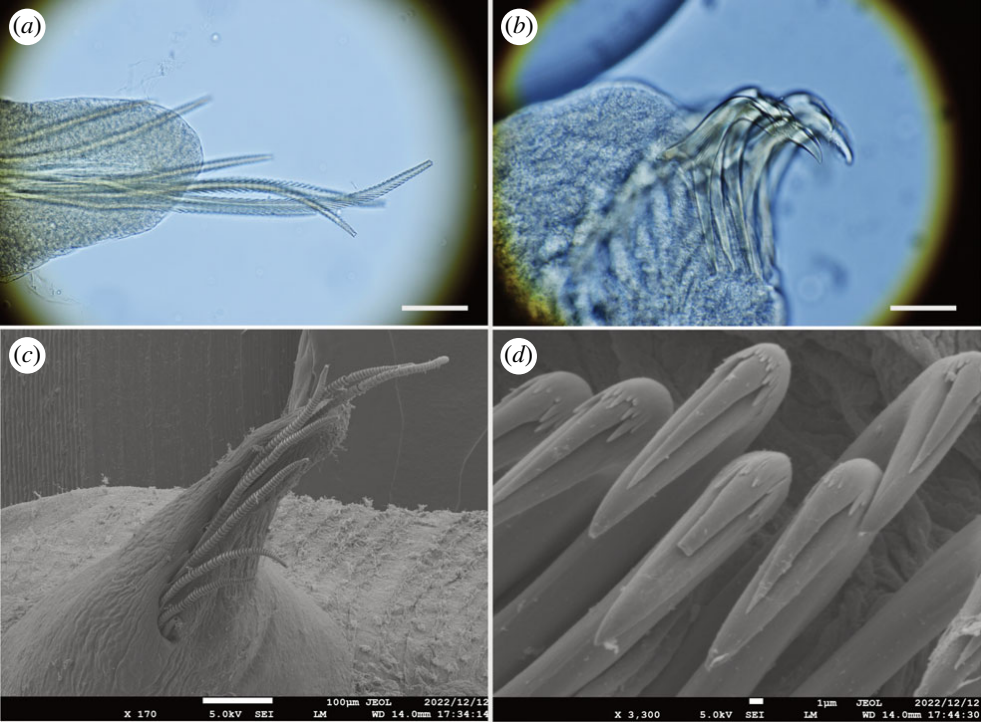
*Polycirrus onibi* sp. nov., (*a*,*b*) holotype (NSMT-Pol H-913); (*c*,*d*) paratype (NSMT-Pol P-914), SEM observation. (*a*) Notochaetae, segment 8; (*b*) neurochaetae, segment 20; (*c*) notochaetae, segment 8; (*d*) neurochaetae, segment 20. Scale bars: (*a*) 100 µm; (*b*) 10 µm.

Zoobank LSID: urn:lsid:zoobank.org:act:E3F23D8D-C700-46F8-B909-B84C033A8317

*Material examined*. Holotype (NSMT-Pol H-913): complete, 24 mm in length, 3 mm in width, 56 segments, in front of Sugashima Marine Biological Laboratory, Mie, Japan (34.4845° N, 136.8756° E), 1 m depth, 28 May 2022, collected by N.J., some parapodia used for DNA extraction. Paratypes: two specimens (NSMT-Pol P-914), complete, 12–21 mm in length, 2 mm in width, 27–49 segments, collected with holotype, used for SEM observation; two specimens, (NSMT-Pol P-915), complete, 16–22 mm in length, 3 mm in width, 60–62 segments, Notojima, Ishikawa, Japan (32.12° N, 137.33° E), 1 m depth, 13 November 2022, collected by T.H., S.K., Y.M., Y.Oh. and K.O., some parapodia used for DNA extraction and SEM observation.

*Diagnosis*. *Polycirrus* with transparent body wall, tentacles with subterminal red spots, mid-ventral groove from segment 3, notochaetae on segments 3–14, neurochaetae on segment 15 and following segments, type II uncini, nephridial papillae in anterior area of parapodia on segments 3–14.

*Description*. Body wall transparent in life and whitish after fixation with ethanol (figures 2*a* and 3), slightly broader until segment 7, then gradually tapering to narrower uniformly cylindrical posterior body. Dorsum anteriorly tessellated. Venter anteriorly with mid-ventral groove and ventro-lateral pads (figure 3*d*); pads tessellated, extending posteriorly from segment 3 to segment 13. Mid-ventral groove from segment 3.

Prostomium fused with base of upper lip and unclear boundary in dorsal and ventral sides. Buccal tentacles white in life and after fixation (figure 3*c,d*), two types: long and thin tentacles uniformly cylindrical; long and thick ones deeply grooved. Red spots present subterminally on both types of tentacles in life, faded after fixation with ethanol. Peristomium forming lips. Upper lip trefoiled with lateral blindly ending enclosed diverticula, margin of medial lobe convoluted; oral surface glandular, ciliated, with grooves leading to mouth. Inner lower lip oblong, smooth; outer region flat, shield-like, rounded and pointing toward mouth, ridged and grooved, extending posteriorly to segment 2. Achaetous segments visible dorsally but obscured by expanded outer lower lip ventrally.

Notopodia from segment 3, ending in segment 14; distinctly elongate, rectangular, first pair slightly shorter, bilobed, prechaetal and postchaetal lobes same shape, digitiform. Two types of notochaetae within a chaetiger (figure 4*a*,*c*); first type pinnate; second type smooth, narrowly winged, uniformly tapered, shorter than pinnate chaetae. Neuropodia beginning from segment 15; uncini with long neck and concave base (Type II) (figure 4*b*), teeth above main fang arranged in double transverse series (MF:1:4–10) (figure 4*d*), enlarged median tooth above main fang present, subrostral process absent.

Nephridial papillae present on anterior area of parapodia (figure 3*d*), segments 3–14.

Pygidium rounded, red pigmentation presents in life (figure 3*a*), faded after preservation.

Etymology. The new species name derives from the Japanese yōkai ‘*onibi*’. Onibi represents the soul of a deceased human or animal, manifested as a floating blue flame. It is often equated with the Will-o'-the-wisp. The blue–purple bioluminescence is reminiscent of this yōkai.

*Distribution and habitat*. Only known from the collection sites, Sugashima (Mie, the northwestern Pacific Ocean) and Notojima (Ishikawa, the Sea of Japan); 1 m depth; muddy sediments or inside cracks of rocks.

*Remarks*. This species resembles *Polycirrus disjunctus* Hutchings & Glasby, 1986 [22] in having similar shape notopodial pre- and post-chaetal lobes, neurochaetae on segment 15 and type II uncini. The new species can be discriminated by the arrangement of notochaetae and nephridial papillae. While notochaetae are present on segments 3–14 and nephridial papillae on the anterior side of parapodia in *P*. *onibi* sp. nov., notochaetae present on segments 3–13 and nephridial papillae present on the posterior side of parapodia in *P. disjunctus*.

Two species, *Polycirrus medius* Hessle, 1917 [34] and *P*. *nervosus* Marenzeller, 1884 [33] have been described from Japan [32–34]. *Polycirrus medius* has been collected from Sagami Sea, approximately 30–40 m depth, in muddy sediment. The new species differs from *P*. *medius* by the presence of neurochaetae beginning about the last notochaetigerous segment (well before the last notochaetigerous segment in *P*. *medius*), by the presence of mid-ventral groove from segment 3 (from segment 4 or 5 in *P*. *medius*), by the presence of ventral pads on segments 3–13 (on segments 2–8 in *P*. *medius*), by rounded lower lip (oblong in *P*. *medius*), and by the presence of one type of notochaetae on segments 3–14 (two types of notochaetae on segments 3–17 in *P*. *medius*). *Polycirrus nervosus* has been collected from the east coast of Enoshima Island, intertidal, rocky shore. The new species differs from *P*. *nervosus* by the presence of ventral groove from segment 3 (segment 4 in *P*. *nervosus*), by the number of notochaetigerous segments (12 in *P*. *onibi* sp. nov., 42 in *P*. *nervosus*), by the absence of leaf-shaped tentacles (present in *P*. *nervosus*), by the shape of upper lip (trefoiled in *P*. *onibi* sp. nov., convoluted in *P*. *nervosus*), by the presence of two types of notochaetae (one type in *P*. *nervosus*), and by the presence of type II uncini (type I uncini in *P*. *nervosus*).

*Polycirrus ikeguchii* Jimi, sp. nov.

**Figure 5.**
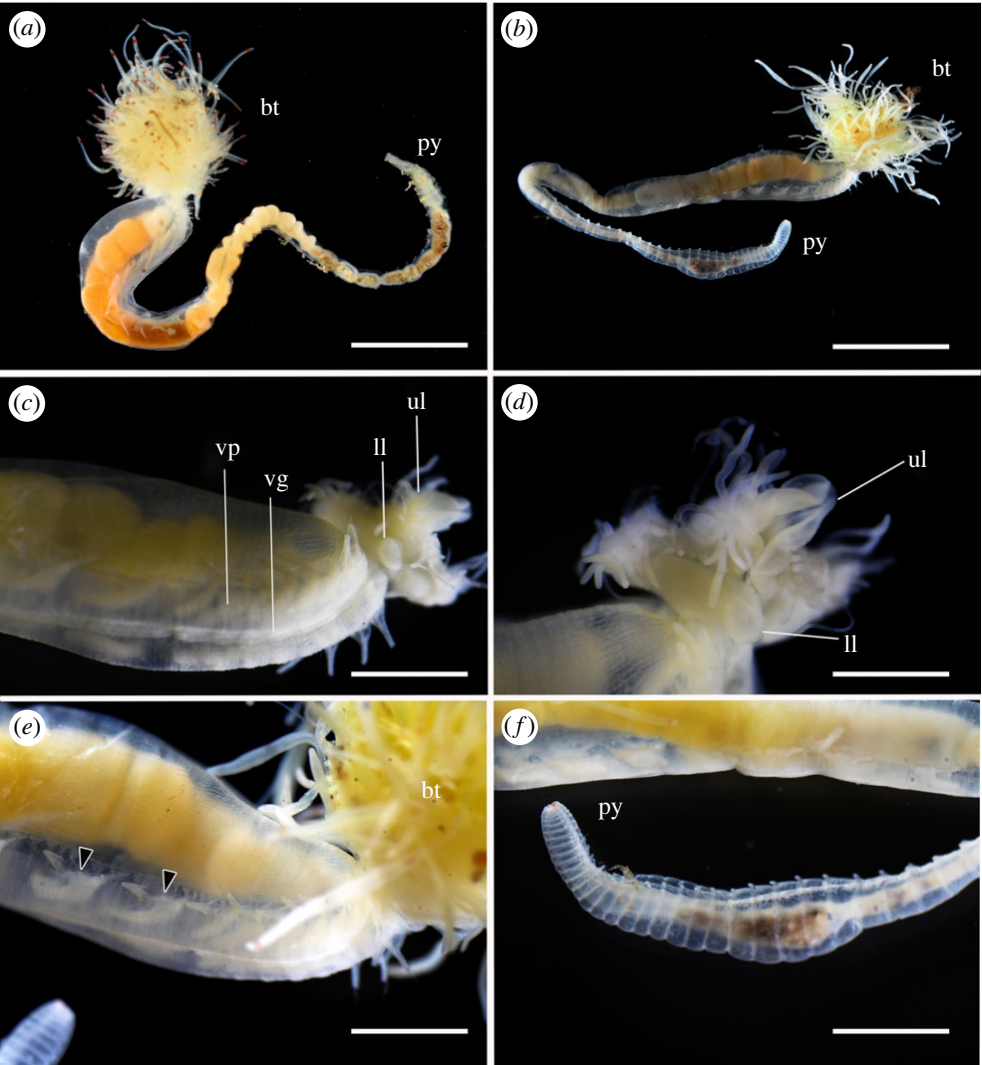
*Polycirrus ikeguchii* sp. nov., holotype (NSMT-Pol H-916): (*a*) live specimen; (*b*–*f*) fixed specimen. (*a*) Whole body, lateral view; (*b*) whole body, lateral view; (*c*) anterior end, lateral view; (*d*) anterior end, ventral view. Scale bars: (*a*,*b*) 5 mm; (*c*) 1 mm; (*d*) 0.5 mm; (*e*,*f*) 1 mm. bt, buccal tentacles; ll, lower lip; py, pygidium; ul, upper lip; vg, ventral groove; vp, ventral pad. Arrow heads indicate nephridial papillae.

**Figure 6.**
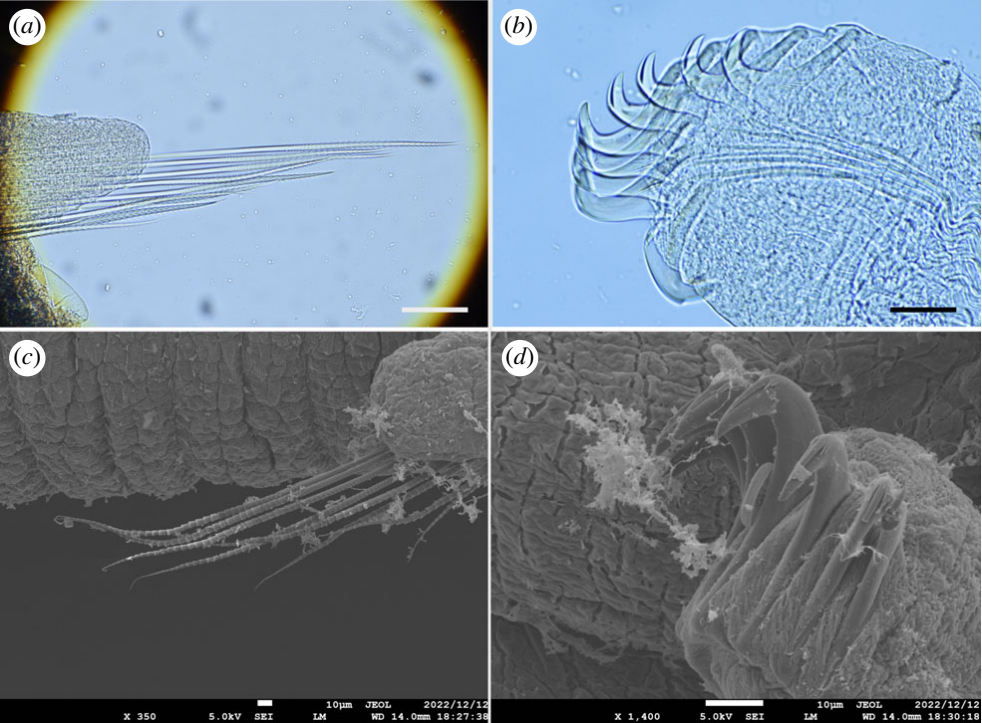
*Polycirrus ikeguchii* sp. nov., (*a*,*b*) holotype (NSMT-Pol H-916); (*c*,*d*) paratype (NSMT-Pol P-917), SEM observation. (*a*) Notochaetae, segment 8; (*b*) neurochaetae, segment 20; (*c*) notochaetae, segment 8; (*d*) neurochaetae, segment 20. Scale bars: (*a*) 100 µm; (*b*) 10 µm.

[Japanese name: Ikeguchi-fusa-gokai]

(figures 2, 5 and 6)

*Polycirrus* sp. ISK. Kanie *et al*. [40]

Zoobank LSID: urn:lsid:zoobank.org:act:EC07BD53-C4AB-40AC-9FDB-8F888500FFDF

*Material examined*. Holotype (NSMT-Pol H-916): complete, 31 mm in length, 1 mm in width, 64 segments, off Shirawara Coast, Tottori, Japan (35.65° N, 134.50° E), 15 m depth, 21 Oct. 2022, collected by AT and YOt, some parapodia used for DNA extraction. Paratype (NSMT-Pol P-917): complete, 25 mm in length, 1 mm in width, 60 segments, off Cape Haneo, Tottori, Japan (35.60° N, 134.33° E), 5 m depth, 20 October 2022, collected by A.T. and Y.Ot., used for SEM observation. Additional specimen: Notojima, Ishikawa, Japan (32.12° N, 137.33° E), 1 m depth, 7 October 2018, collected by T.H., S.K., Y.M., Y.Oh. and K.O., whole body was used for molecular analysis in Kanie *et al*. [40].

*Diagnosis*. *Polycirrus* with transparent body wall, tentacles with subterminal red spots, mid-ventral groove from segment 2, notochaetae on segments 3–19, neurochaetae on segment 17 and following segments, type II uncini, nephridial papillae in anterior area of parapodia, on segments 3–17.

*Description*. Body transparent in life and after fixation with ethanol (figure 5), slightly broader until segment 19, then gradually tapering to narrower uniformly cylindrical posterior body. Dorsum anteriorly tessellated. Venter anteriorly with mid-ventral groove and indistinct ventro-lateral pads (figure 5*c*); pads tessellated, extending posteriorly from segment 3 to segment 19. Mid-ventral groove from segment 2.

Prostomium fused with base of upper lip, and boundary hard to identify in dorsal and ventral sides. Buccal tentacles white in life and after fixation (figure 5*a*,*b*), two types: long and thin tentacles uniformly cylindrical; long and thick ones deeply grooved. Red spots present subterminally on both types of tentacle in life (figure 5*a*,*b*), faded after fixation with ethanol. Peristomium forming lips (figure 5*c*,*d*). Upper lip trefoiled with lateral blindly ending enclosed diverticula, margin of medial lobe convoluted; oral surface glandular, ciliated, with grooves leading to mouth. Inner lower lip oblong, smooth; outer region flat, shield-like, rounded and pointing toward mouth, ridged and grooved, extending posteriorly to segment 2. Achaetous segments visible dorsally but obscured by expanded outer lower lip ventrally.

Notopodia from segment 3, ending in segment 19; distinctly elongate, rectangular, first pair slightly shorter, bilobed, prechaetal and postchaetal lobes same shape, digitiform. Two types of notochaetae within a chaetiger (figure 6*a*); first type pinnate; second type smooth, narrowly winged, uniformly tapered, shorter than pinnate chaetae. Neuropodia beginning from segment 17; uncini with long neck and concave base (Type II) (figure 6*b*), teeth above main fang arranged in one transverse series (MF:6–10) (figure 6*d*), enlarged median tooth above main fang present, subrostral process absent.

Nephridial papillae present on anterior area of parapodia (figure 5*e*), on segments 3–19.

Pygidium rounded, red pigmentation presents in life (figure 5*f*), faded after preservation.

*Etymology*. The species is named after Mr Shinichiro Ikeguchi. He is the former deputy director of the Notojima Aquarium, and he contributed to the discovery of the luminescence phenomenon of this species.

*Distribution and habitat*. Only known from the collection sites, off Shirawara Coast and off Cape Haneo (Tottori, the Sea of Japan) and Notojima (Ishikawa, the Sea of Japan); 1–15 m depth; muddy sediments or inside cracks of rocks.

*Remarks*. This species resembles *Polycirrus aquila* Caullery, 1944 [43] and *P*. *coccineus* Grube, 1870 [44] in the type of notochaetae and neurochaetae, having pinnate notochaetae and type II uncini. The new species differs from *P*. *aquila* by the presence of two types of notochaetae (one type in *P*. *aquila*) and by the presence of notochaetae and nephridial papillae on segments 3–19 (on segments 3–16 in *P*. *aquila*). The new species differs from *P*. *coccineus* by the presence of notochaetae on segments 3–16 (on segments 3–22 in *P*. *coccineus*), by the presence of two types of notochaetae (one type in *P*. *coccineus*), by the arrangement of uncini (MF:6–10 in *P*. *ikeguchii* sp. nov., MF:1:7–8 in *P*. *coccineus*), and by the presence of nephridial papillae on segments 3–16 (on segments 3–9 in *P*. *coccineus*).

*Polycirrus ikeguchii* sp. nov. can be discriminated from other known species of Japan (*P. medius*, *P*. *nervosus* and *P*. *onibi* sp. nov.) by the number of notochaetigerous segments (17 in *P*. *ikeguchii* sp. nov., 15 in *P*. *medius*, 42 in *P*. *nervosus*, 12 in *P*. *onibi* sp. nov.), by the presence of nephridial papillae on segments 3–19 (3–8 in *P*. *medius*, 3–10 in *P*. *nervosus*, 3–14 in *P*. *onibi* sp. nov.), neurochaetae beginning around the last notochaetigerous segment (well before the last notochaetigerous segment in *P*. *nervosus*, around the last notochaetigerous segment in *P*. *medius* and *P*. *onibi* sp. nov.), and type II uncini (type I in *P*. *nervosus*, type II in *P*. *medius* and *P*. *onibi* sp. nov.).

DNA barcode (Genbank No. LC601006) and RNA-seq data (Genbank No. DRX256761, DRX256762) provided in Kanie *et al*. [40] were based on this species.

*Polycirrus aoandon* Jimi, sp. nov.

**Figure 7.**
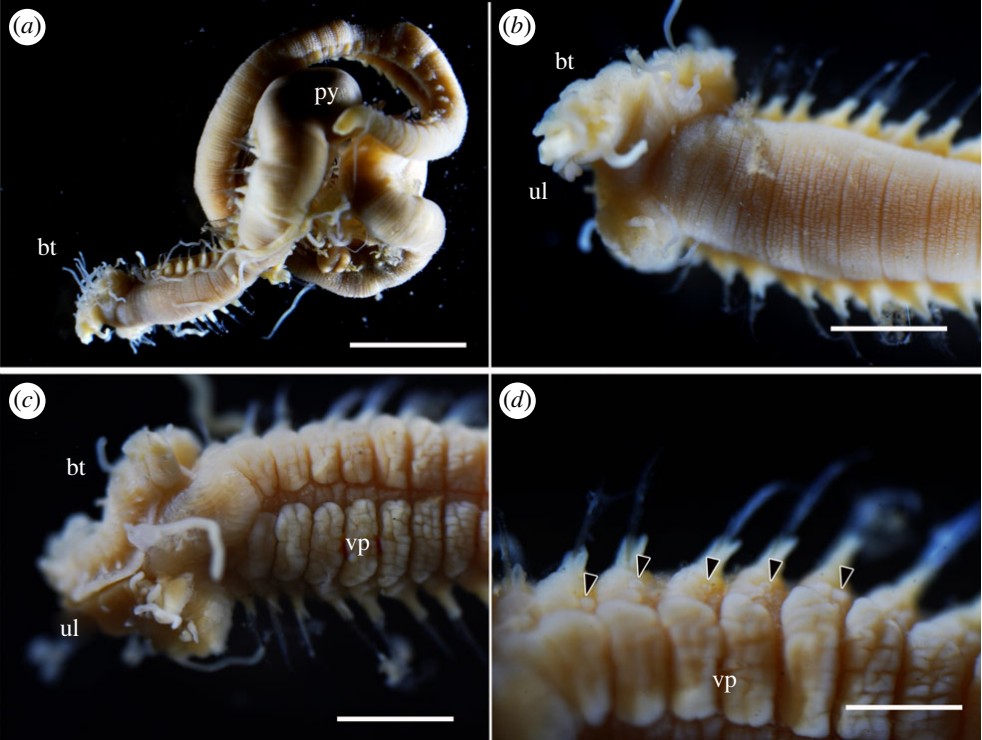
*Polycirrus aoandon* sp. nov., holotype (NSMT-Pol H-918), fixed specimen. (*a*) Whole body, lateral view, fixed specimen; (*b*) anterior end, dorsal view; (*c*) anterior end, dorsal view; (*d*) enlarged view of anterior segments, ventral view. Scale bars: (*a*) 2 mm; (*b*,*c*) 1 mm; (*d*) 0.5 mm. bt, buccal tentacles; py, pygidium; vp, ventral pads. Arrow heads indicate nephridial papillae.

**Figure 8.**
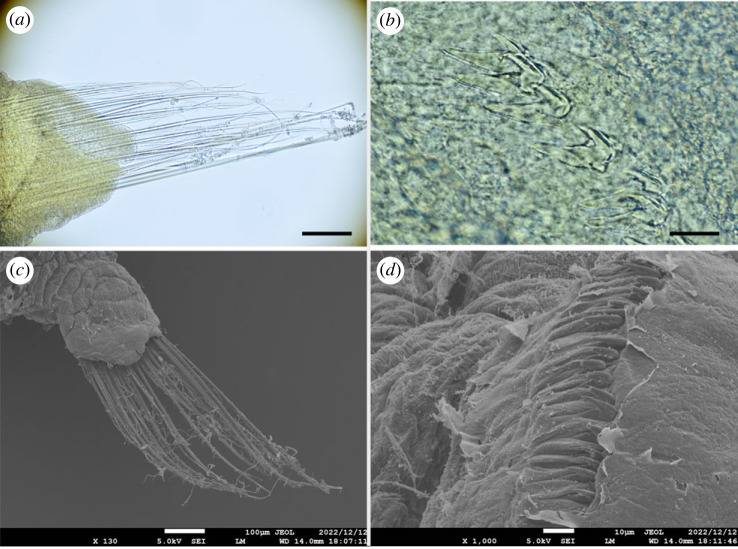
*Polycirrus aoandon* sp. nov., holotype (NSMT-Pol H-918). (*a*) Notochaetae, segment 8; (*b*) neurochaetae, segment 60; (*c*) notochaetae, segment 8; (*d*) neurochaetae, segment 60. Scale bars: (*a*) 100 µm; (*b*) 20 µm.

[Japanese name: Aoandon-fusa-gokai]

(figures 2, 7 and 8)

Zoobank LSID: urn:lsid:zoobank.org:act:089B0747-EEF7-4110-BAA7-AE3E2F1CF589

*Material examined*. Holotype (NSMT-Pol H-918): complete, in front of Sugashima Marine Biological Laboratory, Mie, Japan (34.4845° N, 136.8756° E), 2 m depth, 28 May 2022, collected by N.J. Paratype (NSMT-Pol P-919): same locality as holotype, 1 m depth, 16 December 2022, collected by N.J.

*Diagnosis*. *Polycirrus* with orange body wall, tentacles without subterminal red spots, mid-ventral groove from segment 3, notochaetae on segments 3–56, neurochaetae on segment 16 and following segments, type I uncini, nephridial papillae in anterior area of parapodia on segments 3–14.

*Description*. Body 45 mm in length, 2 mm in width, 80 segments, orange in life and whitish after fixation with ethanol (figures 2*b* and 7), slightly broader until segment 42, then gradually tapering to narrower uniformly cylindrical posterior body. Dorsum anteriorly tessellated (figure 7*b*). Venter anteriorly with mid-ventral groove and developed ventro-lateral pads (figure 7*c*); pads tessellated, extending posteriorly from segment 3 to segment 16. Mid-ventral groove from segment 3.

Prostomium fused with base of upper lip and boundary hard to identify in dorsal and ventral sides. Buccal tentacles orange in life (figure 2*b*) and whitish after fixation (figure 7*a*), red spots absent, two types: long and thin tentacles uniformly cylindrical; long and thick ones deeply grooved. Peristomium forming lips. Upper lip trefoiled with lateral blindly ending enclosed diverticula, margin of medial lobe convoluted; oral surface glandular, ciliated, with grooves leading to mouth. Inner lower lip oblong, smooth; outer region flat, shield-like, rectangular, ridged and grooved, extending posteriorly to segment 2. Achaetous segments visible dorsally but obscured by expanded outer lower lip ventrally.

Notopodia from segment 3, ending in segment 56; distinctly elongate, rectangular, first pair slightly shorter, bilobed, prechaetal and postchaetal lobes same shape, triangular. Notochaetae one type, smooth (figure 8*a*,*c*). Neuropodia beginning from segment 16; uncini with short neck and straight base (Type I) (figure 8*b*), teeth above main fang arranged in one transverse series (MF:3–5) (figure 8*d*), enlarged median tooth above main fang present, subrostral process absent.

Nephridial papillae present on ventral base of parapodia (figure 7*d*), on segments 3–14.

Pygidium rounded, pigmentation unknown in life and absent after fixation with ethanol.

*Etymology*. The new species name derives from the Japanese yōkai ‘*Aoandon*’. Aoandon carries a blue (= *Ao* in Japanese) lantern (= *andon* in Japanese). The blue–purple bioluminescence is reminiscent of this yōkai.

*Distribution and habitat*. Only known from the type locality, Sugashima (Mie, the northwestern Pacific Ocean); 1–2 m depth; muddy sediments under rocks.

*Remarks*. This species resembles *Polycirrus purpureus* Schmarda, 1861 [45] described from the shallow water of Jamaica, in having many notochaetigerous segments, smooth notochaetae, nephridial papillae present on the ventral base of notopodia, and neurochaetae without subrostral process [32,45]. The new species differs from *P*. *purpureus* by the presence of 56 notochaetigerous segments (82–84 notochaetigerous segments in *P*. *purpureus*), by the presence of neurochaetae from segment 16 (from segment 14 on *P*. *purpureus*), by the presence of ventro-lateral pads on segments 3–16 (on segments 3–12 in *P*. *purpureus*), and by the shape of notochaetal lobes (notopodial pre- and post-chaetal lobes of similar shape in *P*. *aoandon* sp. nov., postchaetal lobe longer than prechaetal lobe in *P*. *purpureus*). *Polycirrus aoandon* sp. nov. can be discriminated from other known species of Japan (*P. medius*, *P*. *nervosus*, *P*. *onibi* sp. nov. and *P*. *ikeguchii* sp. nov.) by the having as many as 53 notochaetigerous segments (17 in *P*. *ikeguchii* sp. nov., 15 in *P*. *medius*, 42 in *P*. *nervosus*, 12 in *P*. *onibi* sp. nov.), by the presence of ventral pads on segments 3–16 (2–8 in *P*. *medius,* 3–11 in *P*. *nervosus*, 3–13 in *P*. *onibi* sp. nov., 3–19 in *P*. *ikeguchii* sp. nov.), by the presence of nephridial papillae on segments 3–14 (3–8 in *P*. *medius*, 3–10 in *P*. *nervosus*, 3–14 in *P*. *onibi* sp. nov., 3–19 in *P*. *ikeguchii* sp. nov.), by neurochaetae without subrostral process (present in *P*. *nervosus*, absent in *P*. *ikeguchii* sp. nov., *P*. *onibi* sp. nov. and *P*. *medius*).

## 5. Phylogenetic analysis

**Figure 9.**
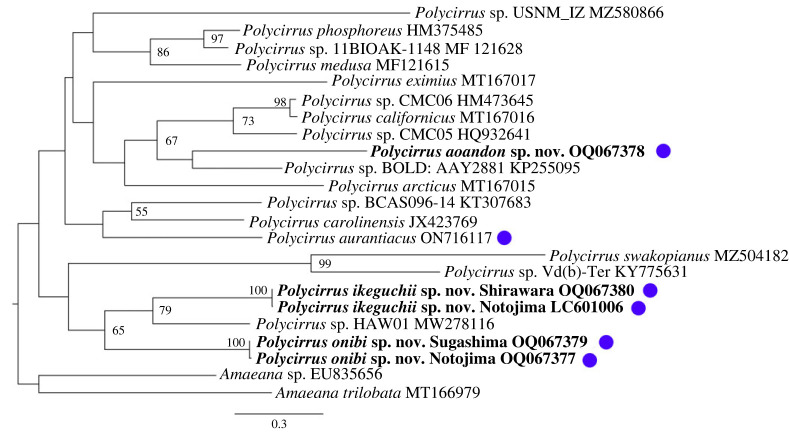
Maximum-likelihood phylogenetic tree of *Polycirrus* species based on partial COI sequences in the barcoding region. *Amaeana* sp. and *A. trilobata* were used as an outgroup. Nodal supports (bootstrap support value in % from 1000 replicates) higher than 50% are indicated on each branch. Blue circles indicate species which have bioluminescent ability. GenBank accession numbers follow the species names.

The resulting phylogenetic tree (figure 9) shows how the three new species of *Polycirrus* are grouped within the *Polycirrus* clade. *Polycirrus aoandon* sp. nov. forms a clade with *Polycirrus* sp. BOLD:AAY2881. *Polycirrus onibi* sp. nov. forms a clade with *P*. *ikeguchii* sp. nov.—*Polycirrus* sp. HAW01 clade with moderate support.

### 5.1. Key to species of Japanese *Polycirrus*

1. Neurochaetae beginning around last notochaetigerous segment… 2

– Neurochaetae beginning well before last notochaetigerous segment… 3

2. Notochaetae on segments 3–14, neurochaetae from segment 15 and following segments… *P*. *onibi* sp. nov. This study [Sea of Japan (Notojima) and the North Westen Pacific Ocean (Sugashima), subtidal]

– Notochaetae on segments 3–19, neurochaetae from segment 17 and following segments… *P*. *ikeguchii* sp. nov. This study [subtidal area of Sea of Japan (Notojima and Shirawara)]

3. Uncini with short neck and straight base (Type I)… 4

– Uncini with long neck and concave base (Type II)… *P*. *medius* [34] [Sagami Sea, subtidal]

4. Uncini with subrostral process… *P*. *nervosus* [33] [Enoshima, intertidal]

– Uncini without subrostral process… *P*. *aoandon* sp. nov. This study [Sugashima Island, subtidal]

## 6. Discussion

Research on the genus *Polycirrus* has been on the rise in recent years, with 27 species being described since the year 2000. The increased interest in taxonomic research of this genus can be attributed to the global review conducted by Glasby and Hutchings in [32]. However, there has been a lack of taxonomic studies on *Polycirrus* in Japan since the description of *P*. *nervosus* by Marenzeller [33] and *P*. *medius* by Hessle [34]. This study represents a significant step forward in the study of *Polycirrus* diversity in Japan, particularly with the discovery of the new species *P*. *onibi* sp. nov., which is found in the shallow subtidal zone of the Sea of Japan (Notojima) and the North Western Pacific Ocean (Sugashima), and is therefore easily accessible for collection in ecological and other studies.

Phylogenetic molecular analysis has confirmed that the three species described in this study are contained within the genus *Polycirrus* (figure 9). However, the overall bootstrap support for this phylogenetic tree is low*.* Further sampling of operational taxonomic units is necessary to determine the evolution of these species. It has also been revealed that *P. onibi* sp. nov. and *P. ikeguchii* sp. nov., which have morphological similarities, are closely related. It is possible that the type of uncini (type II) is a synapomorphy reflecting their phylogenetic relationships.

The ability to emit light occurs in various types of annelids, and it is thought to have been independently acquired multiple times [6]. The luminescence of Terebelliformia, the suborder to which *Polycirrus* belongs, has been poorly studied, with only a few reports on the genera *Thelepus* and *Polycirrus* [8,37–40]. In this study, we compare the luminescence between two species of the same genus for the first time and find no apparent differences in their luminescence patterns or colours. Molecular analysis revealed that *P*. *aoandon* sp. nov. and *P*. *onibi* sp. nov. are not closely related, but their shared trait of luminescence suggests the possibility that luminescence is a common trait within the entire genus. However, since we only compared individual specimens, it is not possible to draw a definitive conclusion. It will be possible to elucidate whether there are differences in bioluminescence between different species by conducting further additional experiments in the future. Further research will help us better understand the phenomenon of bioluminescence in *Polycirrus*, which is known for its characteristic short-wavelength luminescence. Bioluminescence has also been observed in other genera in Terebelliformia, such as *Thelepus*, but the wavelength of this luminescence (508 nm) differs from that of *Polycirrus* (444 nm) [8,40]. In order to understand how bioluminescence has been acquired within Terebelliformia, further study on closely related taxa to *Polycirrus*, such as *Amaeana,* is necessary.

The ecological significance of bioluminescence in *Polycirrus* has been discussed in previous studies [39,40], with some suggesting that it may function as a warning to predators. The species discovered in this study are also known to live buried in mud or in crevices of rocks. Therefore, we opined that they cannot effectively communicate with their surroundings through luminescence unless they are dug up to expose themselves. This indicates that the luminescence of this genus may only be useful in times of emergency situations when their bodies are exposed. In our observations, we found that the organism emitted light when stimulated with a pinprick (figure 2, electronic supplementary material, videos S1 and S2). This suggests that the *Polycirrus* worm is emitting light in response to external stimuli, which supports the hypothesis that it is exhibiting a defensive response to potential threats.

## Data Availability

The videos depicting observation of bioluminescence are appended as appendix videos [46]. Genetic data can be obtained from Genbank (Accession nos. OQ067377–OQ067380: https://www.ncbi.nlm.nih.gov/genbank).
